# Biophysical Studies on Interactions and Assembly of Full-size E3 Ubiquitin Ligase: SUPPRESSOR OF CYTOKINE SIGNALING 2 (SOCS2)-ELONGIN BC-CULLIN 5-RING BOX PROTEIN 2 (RBX2)*

**DOI:** 10.1074/jbc.M114.616664

**Published:** 2014-12-11

**Authors:** Emil Bulatov, Esther M. Martin, Sneha Chatterjee, Axel Knebel, Satoko Shimamura, Albert Konijnenberg, Clare Johnson, Nico Zinn, Paola Grandi, Frank Sobott, Alessio Ciulli

**Affiliations:** From the ‡ Division of Biological Chemistry and Drug Discovery, College of Life Sciences, and; the ‖ Medical Research Council Phosphorylation and Ubiquitylation Unit, College of Life Sciences, Sir James Black Center, University of Dundee, Dundee DD1 5EH, Scotland, United Kingdom,; the § Department of Chemistry, University of Cambridge, Cambridge CB2 1EW, United Kingdom,; the ¶ Department of Chemistry, University of Antwerp, 2020 Antwerp, Belgium, and; ** Cellzome GmbH, Meyerhofstrasse 1, 69117 Heidelberg, Germany

**Keywords:** Biophysics, E3 Ubiquitin Ligase, Isothermal Titration Calorimetry (ITC), Mass Spectrometry (MS), Post-translational Modification (PTM), Protein Assembly, Protein Complex, Protein-Protein Interaction, Cullin

## Abstract

The multisubunit cullin RING E3 ubiquitin ligases (CRLs) target post-translationally modified substrates for ubiquitination and proteasomal degradation. The suppressors of cytokine signaling (SOCS) proteins play important roles in inflammatory processes, diabetes, and cancer and therefore represent attractive targets for therapeutic intervention. The SOCS proteins, among their other functions, serve as substrate receptors of CRL5 complexes. A member of the CRL family, SOCS2-EloBC-Cul5-Rbx2 (CRL5^SOCS2^), binds phosphorylated growth hormone receptor as its main substrate. Here, we demonstrate that the components of CRL5^SOCS2^ can be specifically pulled from K562 human cell lysates using beads decorated with phosphorylated growth hormone receptor peptides. Subsequently, SOCS2-EloBC and full-length Cul5-Rbx2, recombinantly expressed in *Escherichia coli* and in Sf21 insect cells, respectively, were used to reconstitute neddylated and unneddylated CRL5^SOCS2^ complexes *in vitro*. Finally, diverse biophysical methods were employed to study the assembly and interactions within the complexes. Unlike other E3 ligases, CRL5^SOCS2^ was found to exist in a monomeric state as confirmed by size exclusion chromatography with inline multiangle static light scattering and native MS. Affinities of the protein-protein interactions within the multisubunit complex were measured by isothermal titration calorimetry. A structural model for full-size neddylated and unneddylated CRL5^SOCS2^ complexes is supported by traveling wave ion mobility mass spectrometry data.

## Teaser

**Background:** The component subunits of CRL E3 ligases assemble into specific complexes.

**Results:** Components of CRL5^SOCS2^ were identified from human cell lysate, the full-size complex was reconstituted *in vitro*, and protein-protein interactions were biophysically characterized.

**Conclusion:** CRL5^SOCS2^ components exist in a monomeric state, and proposed structural models are supported by ion mobility mass spectrometry.

**Significance:** We provide structural insights into the assembly of full-size CRL5^SOCS2^ that can aid development of small molecules targeting CRL complexes.

## Introduction

Cullin RING E3 ubiquitin ligases (CRLs)^3^ represent the largest known family of E3 enzymes in the ubiquitin-proteasome system (>200 CRLs of a total of >600 E3 enzymes) and play a significant role in cancer and other diseases (1). In some types of cells, up to 20% of the proteasome-dependent degradation is mediated by CRLs (2). Assembly of the multisubunit CRLs was initially reported for the archetypal Skp1-Cul1-Rbx1 complex (3). CRLs use modular subunit organization consisting of interchangeable adaptors (Skp1, elongin B, elongin C, DDB1, and BTB), substrate receptors (F box, SOCS box, DCAF, and BTB), cullin scaffolds (cullin 1–7), and RING domain proteins (Rbx1 and Rbx2) to enable the assembly of a large number of functionally diverse E3 ligase complexes (1, 4, 5).

The N-terminal domain (NTD) of cullin proteins consists of three five-helix bundles (“cullin repeats”) that form a long stalk architecture, and a globular C-terminal domain (CTD, or “cullin homology domain”). Cullin NTD recruits variable substrate receptors either directly or via an adaptor protein, whereas cullin CTD serves as a docking site for RING domain proteins that in turn recruit a cognate ubiquitin-loaded E2 (6). RING domain proteins contain a distinct Zn^2+^-binding domain characterized by a canonical RING motif. Selection of the substrate receptors for a particular CRL occurs through a specific receptor LP*X*P motif that forms a minor yet crucial supplementary interaction with cullin NTD (7, 8).

SOCS2 is a member of the SOCS box protein family that, in association with the adaptor elongin B-elongin C complex (EloBC), cullin 5 scaffold, and Rbx2, constitutes a CRL5^SOCS2^ E3 ligase. SOCS2 contains three structural domains: a conserved C-terminal SOCS box domain that binds to adaptor EloBC; a central SH2 domain mediating recruitment of phosphorylated tyrosine-containing sequence of the substrate; and a variable N-terminal region that facilitates interaction with the substrate. CRL5^SOCS2^ negatively regulates growth hormone signaling by targeting growth hormone receptor (GHR) for ubiquitination and proteasomal degradation (9). Phosphorylated tyrosine 595 serves as the key structural determinant of GHR recognition by the SH2 domain of SOCS2 (10). Crystal structures of SOCS2-EloBC (PDB code 2C9W) (11) and SOCS2-EloBC-Cul5_NTD_ (PDB code 4JGH) (12) describe structural features of the protein-protein interfaces within these left-arm complexes. However, the details of assembly of the full-size CRL5^SOCS2^ E3 complex both *in vivo* and *in vitro* remain missing.

In recent years, interest in studying the structure, function, and assembly of CRLs has been growing, notably driven in part by their potential role as drug targets in a number of human diseases (13–16). However, only a few studies have investigated the full-size CRL complexes biophysically, primarily due to difficulties in obtaining some of the protein components recombinantly, in particular full-length cullins. Furthermore, large heteromeric protein complexes such as CRLs are notoriously difficult to crystallize into diffraction quality crystals. Therefore, it seems promising to engage the strengths of diverse biophysical methods in order to facilitate characterization of both the individual subunits and the full-size complexes as well as to provide a means for examining their association and interactions.

Here, we show that all components of the CRL5^SOCS2^ could be pulled down from a cell lysate via SOCS2-mediated recognition of the phosphorylated GHR_pY595 peptide immobilized on beads. The full-length E3 ligase complex was then reconstituted *in vitro* using purified recombinant proteins and characterized biophysically. Investigations of assembly and interactions within the complex were carried out using size exclusion chromatography and multiangle light scattering (SEC-MALS), isothermal titration calorimetry (ITC), and nanoelectrospray traveling wave-ion mobility mass spectrometry (TWIM-MS).

## EXPERIMENTAL PROCEDURES

### 

#### 

##### Pull-down Experiments

Pull-down experiments were performed using biotinylated GHR-derived 11-mer peptides phosphorylated (GHR_pY595) or not (GHR_Y595) on tyrosine 595, harboring an aminohexanoic acid as spacer after the biotin (Biotin-aminohexanoic acid-PVPDpYTSIHIV-amide), and immobilized on high capacity Neutravidin beads. Competition experiments were performed by incubating human K562 total cell lysate with 100 μm non-biotinylated phosphorylated peptide (GHR_pY595) and the immobilized (Biotin-aminohexanoic acid-PVPDpYTSIHIV-amide) beads for 2 h at 4 °C. After washing, bound proteins were eluted with SDS-sample buffer and prepared for tandem mass tags labeling and MS analysis as described previously (17).

##### Protein Expression and Purification

Recombinant human SOCS2 (amino acids 32–198), elongin C (amino acids 17–112), and elongin B (amino acids 1–118) were co-expressed in *Escherichia coli* BL21(DE3) from the pLIC (SOCS2) and pCDF_Duet (EloBC) plasmids (gifts from A. Bullock, Structural Genomics Consortium, Oxford, UK). A starter culture was grown overnight from a single transformant colony using 50 ml of LB medium containing 100 μg/ml ampicillin and 50 μg/ml streptomycin. Starter culture then was used to inoculate 7 liters of LB medium containing 100 μg/ml ampicillin and 50 μg/ml streptomycin. The cells were grown at 37 °C until *A*_600_ ∼0.7 and cooled to 18 °C, and then protein expression was induced with 1 mm isopropyl β-d-1-thiogalactopyranoside for 12 h.

Recombinant human Cul5_NTD_ (N-terminal domain, residues 1–386) was expressed in *E. coli* BL21(DE3) from a pNIC plasmid encoding sequence for Cul5_NTD_, containing His_6_ and FLAG tags at the C-terminal end and a tobacco etch virus (TEV) cleavage site, as described previously (18). Briefly, 50 ml of starter culture was grown overnight using a single transformant colony in LB medium containing 50 μg/ml kanamycin and used to inoculate 2 liters of LB medium supplemented with 50 μg/ml kanamycin. The cells were grown at 37 °C until *A*_600_ ∼0.7 and cooled to 18 °C, and protein expression was induced with 0.5 mm isopropyl β-d-1-thiogalactopyranoside for 12 h.

Recombinant SOCS2-EloBC and Cul5_NTD_ were independently purified using the following protocol. The cell pellets were harvested by centrifugation at 5,000 rpm and 4 °C for 30 min and resuspended in binding buffer (50 mm HEPES, pH 7.5, 500 mm NaCl, 5% glycerol, 0.5 mm TCEP). The supernatant was treated with 10 μg/ml DNase I, 10 mm MgCl_2_ for 30 min and then filtered through a 0.22-μm filter. The sample was applied on a HisTrap column (GE Healthcare), and the resin was washed with wash buffer (50 mm HEPES, pH 7.5, 20 mm imidazole, 500 mm NaCl, 5% glycerol, 0.5 mm TCEP) and then the bound proteins were eluted with an incremental gradient of elution buffer (50 mm HEPES, pH 7.5, 500 mm imidazole, 500 mm NaCl, 5% glycerol, 0.5 mm TCEP). Fractions containing protein were pooled, and the His_6_ tag was cleaved off by overnight dialysis in the presence of TEV protease at 4 °C in binding buffer. The protein was applied to a HisTrap column for a second time, collecting the flow-through, and then concentrated and purified on a HiLoad 16/60 Superdex 75 column with running buffer 25 mm HEPES, pH 7.5, 250 mm NaCl, 0.5 mm TCEP.

Recombinant human Cul5 (amino acids 1–780) and Rbx2 (amino acids 1–113) were co-expressed in Sf21 insect cells using pFastBac^TM^ Dual vector in the Bac-to-Bac® baculovirus expression system. In this vector, Cul5 is N-terminally tagged with a fragment of bacterial PBP5 (Dac tag) (19), which can be removed with TEV protease, as described previously (20).

Bacmids for Dac-TEV-Cul5/Rbx2 were generated in DH10BAC cells and transfected into Sf21 cells, using Cellfectin II® reagent (Invitrogen). The transfected cells were kept for 7 days at 27 °C in Insect Express® medium (Lonza), supplemented with ANTI-ANTI® (Invitrogen). The cells were sedimented, and the virus-containing supernatant was used to infect 150 ml of Sf21 culture at a density of 1.5 × 10^6^ cells/ml. For the expression of RING E3 ligases, we supplemented the Insect Express medium with 5 μm ZnCl_2_. After 5 days the cells, were collected under sterile conditions, and the supernatant was used to infect 2 liters of Sf21 cell culture for 3 days. The Cul5-Rbx2 protein complex was purified using the following procedure. Cells were harvested by centrifugation at 3,500 rpm and 4 °C for 15 min and then resuspended in 25 ml of 50 mm HEPES, pH 7.4, 0.1 mm EGTA, 1 μm ZnCl_2_, 1 mm TCEP, 1 mm Pefabloc®, and 20 μg/ml leupeptin (both from Apollo Scientific) and incubated for 15 min at 4 °C. Cells were sheared using a 50-ml tight fit Dounce homogenizer, and insoluble material was removed by centrifugation at 40,000 rpm and 10 °C for 20 min. To perform Dac affinity purification, the supernatant was gently mixed with ampicillin-Sepharose at room temperature for 50 min. The Sepharose was collected by centrifugation and washed six times in 10 volumes of 50 mm HEPES, pH 7.4, 150 mm NaCl, 0.1 mm EGTA, 1 μm ZnCl_2_, 1 mm TCEP. To recover untagged Cul5-Rbx2, the Sepharose was incubated with TEV protease (50 μg of TEV, 1 ml of Sepharose) overnight at room temperature and drained and washed through Econopac® filter units (Bio-Rad). The Cul5-Rbx2 protein was concentrated and further purified by preparative size exclusion chromatography using HiLoad 16/600 Superdex 200 column (GE Healthcare) with 50 mm HEPES, pH 7.4, 150 mm NaCl, 10% glycerol, 0.5 mm TCEP.

The identity and purity of all obtained proteins was confirmed using denaturing electrospray ionization-MS and SDS-PAGE. All proteins were flash-frozen using liquid nitrogen and stored at −80 °C.

##### Reconstitution of SOCS2-EloBC-Cul5_NTD_ and SOCS2-EloBC-Cul5-Rbx2 Protein Complexes

To form quaternary SOCS2-EloBC-Cul5_NTD_ complex, Cul5_NTD_ and SOCS2-EloBC were mixed together at a 1:1.1 molar ratio and incubated at room temperature for 30 min, following purification of the complex using a HiLoad 16/600 Superdex 75 column in 25 mm HEPES, pH 7.5, 250 mm NaCl, 0.5 mm TCEP.

To form the pentameric complex, Cul5-Rbx2 and SOCS2-EloBC were mixed at a 1:1.1 molar ratio and incubated at room temperature for 30 min. The protein complex was purified using a HiLoad 16/600 Superdex 200 column in 50 mm HEPES, pH 7.4, 150 mm NaCl, 10% glycerol, 0.5 mm TCEP. SDS-PAGE analysis of SOCS2-EloBC, Cul5_NTD_, SOCS2-EloBC-Cul5_NTD_, Cul5-Rbx2, and SOCS2-EloBC-Cul5-Rbx2 is shown in Fig. 3*B*.

##### Formation of NEDD8∼Cul5-Rbx2 Covalent Conjugate

To obtain the conjugate, E1 activating enzyme APP-BP1/UBA3 (1 μm), E2 conjugating enzyme UBE2F (5 μm), NEDD8 (40 μm), and Cul5-Rbx2 (5 μm) were incubated at 37 °C for 1 h in 50 mm HEPES, pH 7.4, 150 mm NaCl, 5 mm DTT, 10 mm MgCl_2_, 0.2 mm ATP. A negative control experiment was performed in the same solution containing 0.1 mm EDTA, with no MgCl_2_ or ATP added. The completion of the neddylation on the Cul5 subunit was confirmed by SDS-PAGE (Fig. 8).

##### SEC-MALS

SEC-MALS experiments were performed using Dionex Ultimate 3000 UHPLC system (Thermo Scientific) with an inline Wyatt miniDAWN TREOS MALS detector and Optilab T-rEXTM refractive index detector. Molar masses spanning elution peaks were calculated using ASTRA version 6.0.0.108 (Wyatt). SEC-MALS data were collected for the following samples: 1) SOCS2-EloBC at 130 μm; 2) Cul5_NTD_ at 110 μm; 3) SOCS2-EloBC-Cul5_NTD_ at 90 μm; 4) Cul5-Rbx2 at 30 μm; 5) SOCS2-EloBC-Cul5-Rbx2 at 35 μm. The experiments were performed using a Superdex 200 10/300 GL column (GE Healthcare) with running buffer 50 mm HEPES, pH 7.5, 150 mm NaCl, 0.5 mm TCEP. The scattering signal was collected at 44, 90, and 136° using λ = 658.5 nm incident light. Resulting data were processed in Microsoft Excel, and peaks were normalized.

##### TWIM-MS

The native TWIM-MS experiments were conducted on a Synapt HDMS G2 instrument (Waters, Milford, MA), which has been described previously (21). Samples following gel filtration were buffer-exchanged into 500 mm aqueous ammonium acetate at pH 7.0, using Micro Bio-Spin P-6 columns (Bio-Rad), at concentrations in the range of 5–10 μm. Aliquots of 3–5 μl were transferred to gold-coated nano-electrospray ionization needles prepared in house. The instrument was tuned to ensure the preservation of non-covalent interactions (22), using the following parameters: capillary, 1.2 kV; sample cone, 40 V; extraction cone, 0.5 V; nanoflow gas pressure, 0.3 bar; trap collision energy, 4.0 V; transfer collision energy, 3.5 V; backing pressure, 4 millibars, trap pressure, 3.4 millibars. For the measurement of the full 148-kDa complex, SOCS2-EloBC-Cul5-Rbx2, the backing pressure was increased to 5 millibars to facilitate the transmission of high *m*/*z* signal. Gas pressure in the ion mobility cell was 3.0 millibars, and helium and N_2_ gas flows were 180 and 90 ml/min, respectively, with a trap bias of 50 V. The traveling wave velocity was 800 m/s with a traveling wave height of 40 V. The data were acquired and processed with MassLynx version 4.1 software (Waters), and drift times were extracted using Driftscope version 2.3 (Waters). The experimental collision cross-sections (CCS) of the protein complexes were determined by calibration with known protein cross-sections determined under native conditions as described previously (23).

##### Calculation of Theoretical CCS

Theoretical CCS values of the protein complexes were calculated from model structures, obtained by docking individual protein subunits together, using the program MOBCAL with both the projection approximation (PA) and the exact hard sphere scattering (EHSS) methods (24, 25). The PDB files were cleaned (*i.e.* by resolving dihedral conflicts and adding missing side chains and removing crystal water molecules) prior to the PA or EHSS calculation. The theoretical CCS was compared with the experimental CCS of the lowest available charge state for that species in the mass spectra, which corresponds to the most native-like structure of the protein complex (Fig. 4, *A–C*, *bottom panels*).

##### ITC

Experiments were conducted using an iTC200 microcalorimeter instrument (GE Healthcare). GHR_pY595 peptide (350 μm, PVPDpYTSIHIV-amide), GHR_Y595 (350 μm, PVPDYTSIHIV-amide), and phosphotyrosine (2 mm, Tyr(P)) were titrated into SOCS2-EloBC (30 μm) at 298 K. Temperature-dependent experiments to study interaction between SOCS2-EloBC and Cul5_NTD_ were performed by titrating Cul5_NTD_ (450 μm) into SOCS2-EloBC (60 μm) at 298, 303, and 308 K. Prior to all titration experiments, sample proteins were dialyzed into 50 mm HEPES, pH 7.5, 250 mm NaCl, 0.5 mm TCEP. Peptides and Tyr(P) were dissolved in the same buffer. Obtained data were analyzed and fitted using the Microcal Origin version 7.0 software package. Binding enthalpy, dissociation constants, and stoichiometry were determined by fitting the data using a one-set-of-site binding model.

##### Molecular Modeling of Protein Complexes

Due to the absence of an Rbx2 crystal structure, its closest homolog, Rbx1, was used for the model construction. The structural model of the SOCS2-EloBC-Cul5-Rbx1 complex was prepared in PyMOL, using the crystal structure of Cul1-Rbx1-Skp1-Skp2 (PDB code 1LDK) (26) as the initial template. To construct the model, SOCS2-EloBC-Cul5_NTD_ (PDB code 4JGH) (12) was superimposed on the template by aligning its Cul5_NTD_ subunit with the Cul1_NTD_ of the template. After that, Cul5_CTD_-Rbx1 (PDB code 3DPL) (27) was aligned with Cul1_CTD_ subunit of the template to generate a model of the full-length E3 ligase. The resulting model of the CRL5^SOCS2^ complex was used to obtain the model for Cul5-Rbx1. To generate a model of the “open” neddylated complexes, NEDD8∼Cul5_CTD_-Rbx1 (PDB code 3DQV) (27) was aligned with the Cul1_CTD_ subunit of the template. Alternatively, to prepare a model of the closed neddylated complexes, NEDD8 was simply added from aligned 3DQV onto the non-neddylated Cul5_CTD_-Rbx1 and SOCS2-EloBC-Cul5-Rbx1.

## RESULTS

### 

#### 

##### Components of CRL5^SOCS2^ E3 Ligase Can Be Pulled Down from Human Cell Lysates Using Phosphopeptide-modified Beads

**FIGURE 1.**
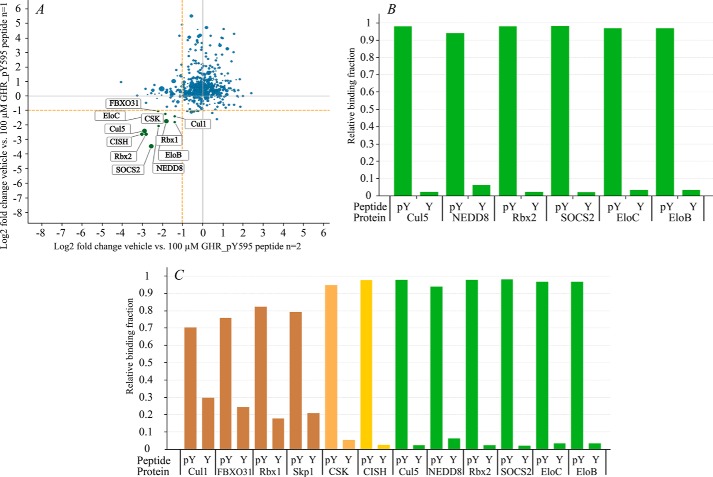
**Components of CRL5^SOCS2^ E3 ligase can be captured from cell lysate using phosphorylated substrate peptide attached to the beads.** *A*, scattered plot of the peptide pull-down experiments after MS analysis: log2 -fold changes of all proteins captured on the phosphorylated GHR_pY595 peptide beads after competition with a 100 μm concentration of the same peptide in the lysate. Each protein is indicated by a *circle*, and the *size* of the *circle* is proportional to the MS1 value. The labeled proteins are at least 50% displaced by the addition of the peptide in both experiments (log2 -fold change ≤1, *orange dotted lines*). *B*, relative binding profile of the components of CRL5^SOCS2^ E3 ligase complex captured on the beads decorated with phosphorylated (*pY*) *versus* non-phosphorylated (*Y*) GHR_(p)Y595 peptide. *C*, binding profiles for other proteins specifically recruited by the beads. Components of the CRL5^SOCS2^ E3 ligase are *colored* in *green*.

Specific subunits of E3 ligase SOCS2, EloB, EloC, Cul5, and Rbx2 are known to function as a CRL5^SOCS2^ complex (28). The SH2 domain of SOCS2 recognizes and specifically binds a GHR sequence containing the phosphorylated tyrosine Tyr(P)^595^. We envisaged that SOCS2 and components of full-length CRL5^SOCS2^ E3 ligase should be amenable for capturing from cell lysate using substrate peptides immobilized on beads. With this aim, we performed pull-down experiments from human K562 cell lysate using beads decorated with both phosphorylated GHR_pY595 (PVPDpYTSIHIV-amide, positive control) and non-phosphorylated GHR_Y595 (PVPDYTSIHIV-amide, negative control) peptides. Mass spectrometry analysis revealed a reproducible and limited set of proteins captured and subsequently displaced by the phosphorylated peptide (Fig. 1*A*, *bottom left corner*). All components of the CRL5^SOCS2^ (SOCS2, EloB, EloC, Cul5, and Rbx2) were among this protein set.

Binding profile (Fig. 1*B*) shows that endogenous components of CRL5^SOCS2^ E3 ligase were only captured by GHR_pY595-beads. In contrast, no significant capturing from cell lysates was observed with non-phosphorylated GHR_Y595-containing beads (Fig. 1*B*). This observation shows that the Tyr(P) residue plays a key role in recognition of the substrate by the CRL^SOCS2^ complex in the cell and that phosphorylation of the peptide is essential for specific interaction with the E3 ligase. Interestingly, we also detected NEDD8 as a protein specifically pulled down by the phosphorylated peptide. NEDD8 is a ubiquitin-like protein that is known to be covalently attached to cullins and acts as a CRL activator by inducing scaffold dynamics and increasing conformational flexibility of the E3 enzyme (27, 29). Identification of NEDD8 suggests that the active neddylated complex is also being pulled down in the assay.

Surprisingly, in addition to the expected CRL5^SOCS2^ complex subunits, we detected subunits of the CRL1^FBXO31^ E3 ligase, namely FBXO31, Rbx1, Cul1, and Skp1 proteins, as being captured by the beads and displaced by the phosphorylated peptide (Fig. 1*A* and Table 1), indicating specific binding. FBXO31 is an F box protein that binds phosphorylated substrates; therefore, it could have been recruited by the GHR_pY595 peptide directly. However, significant recruitment of the four CRL1^FBXO31^ subunits was also observed by the non-phosphorylated GHR peptide (Fig. 1*C*). This would imply a degree of phosphorylation-independent interaction, either directly with the beads or indirectly via binding to the components of the CRL5^SOCS2^ E3 ligase complex.

**TABLE 1:** **Proteins enriched by the phosphorylated GHR_pY595-modified beads** The proteins were specifically captured by the beads and displaced by the phosphorylated GHR peptide.

Protein	UniProt ID	Comments
SOCS2	O14508	Suppressor of cytokine signaling 2. Substrate recognition domain of Cul5^SOCS2^ E3 ligase. Contains SH2 domain that recognizes substrate phosphotyrosine residues.
EloB	Q15370	Transcription elongation factor B.
EloC	Q15369	Transcription elongation factor C. Complex of EloB and EloC (EloBC) serves as adaptor domain of Cul5^SOCS2^ E3 ligase.
Cul5	Q93034	Cullin 5. Scaffold domain of the Cul5^SOCS2^ E3 ligase.
Rbx2	Q9UBF6	RING box protein 2. Contains RING-type zinc finger, recruits E2 conjugating enzyme.
NEDD8	Q15843	Neural precursor cell expressed developmentally down-regulated protein 8. Ubiquitin-like protein, can form covalent conjugate with Cullin that enhances the E3 ligase activity.
CISH	Q9NSE2	Cytokine-inducible SH2-containing protein. Component of SCF E3 ligase, can recognize phosphotyrosine.
Rbx1	P62877	RING-box protein 1. Contains RING-type zinc finger, recruits E2 conjugating enzyme.
CSK	P41240	c-Src kinase. Contains SH2 domain that can recognize phosphotyrosine residues.
Cul1	Q13616	Cullin 1. Scaffold component of SCF E3 ligase.
FBXO31	Q5XUX0	F box-only protein. Substrate recognition component of SCF E3 ligase. Can recognize certain phosphorylated substrates.
Skp1	P63208	S-phase kinase-associated protein 1. Adaptor component of SCF E3 ligase.

Moreover, CSK (C-terminal Src kinase) and CISH (cytokine-inducible SH2-containing) proteins were also recruited (Fig. 1*A* and Table 1). Both CSK and CISH contain the SH2 domain; therefore, both proteins were probably directly recruited by the phosphorylated peptide.

##### SOCS2-EloBC Forms a Weak Interaction with GHR and a Tight Interaction with Cul5_NTD_

**FIGURE 2.**
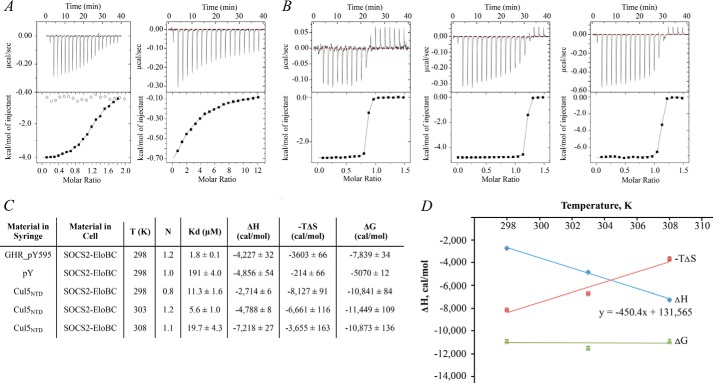
**ITC data demonstrate weak interaction of SOCS2-EloBC with phosphorylated substrate GHR and tight interaction with scaffold Cul5_NTD_.** *A*, ITC titration curves of 350 μm GHR peptides (*left*) and 2 mm Tyr(P) (*right*) into 30 μm SOCS2-EloBC protein at 298 K. Phosphorylated GHR_pY595 peptide (*black squares*) and non-phosphorylated GHR_Y595 (*white circles*) are shown on the *left. B*, titration curves for temperature-dependent ITC of 450 μm Cul5_NTD_ into 60 μm SOCS2-EloBC at 298 (*left*), 303 (middle), and 308 K (*right*). *C*, *table* summarizing data obtained from ITC experiments. *D*, plot demonstrating temperature dependence of thermodynamic parameters and calculation of heat capacity (Δ*C_P_*) for SOCS2-EloBC/Cul5_NTD_ interaction.

To determine the affinity of interaction and the thermodynamic parameters of binding between SOCS2-EloBC and substrate GHR or scaffold Cul5_NTD_, we performed isothermal titration calorimetry experiments (Fig. 2). SOCS2-EloBC binds GHR_pY595 peptide with *K_d_* = 1.8 μm, which is consistent with the previously reported value (11), and binds Tyr(P) with *K_d_* = 191 μm, both at 298 K (Fig. 2*A*). The binding affinity for phosphotyrosine is ∼100-fold weaker than for the phosphorylated peptide, suggesting that other peptide residues make some contribution to interaction with the protein. However, negative control titration using non-phosphorylated GHR_Y595 peptide showed no binding (Fig. 2*A*), reinforcing the key contribution of the phosphate group to substrate binding.

We next determined the affinity of SOCS2-EloBC for Cul5_NTD_ by measuring a *K_d_* = 11 nm for the interaction (Fig. 2*C*). These data are in good agreement with the previously reported *K_d_* of 28 nm (by ITC) (12). In addition, two groups independently reported *K_d_* = 7 nm (by ITC) (30), *K_d_* = 10 nm (by ITC), and *K_d_* = 47 nm (by surface plasmon resonance) (31) for this interaction, albeit using SOCS box domain instead of the whole SOCS2 protein in complex with EloBC.

To test the potential cooperativity of interactions at the GHR/SOCS2-EloBC/Cul5_NTD_ interfaces, we performed titration of GHR_pY595 peptide into SOCS2-EloBC-Cul5_NTD_ and titration of Cul5_NTD_ into GHR_pY595-SOCS2-EloBC complex. No change in the *K_d_* or Δ*H* values was observed in either case, suggesting no cooperativity or cross-talk between these interactions.

The interaction between SOCS2-EloBC and the Cul5 scaffold is high affinity and crucial to the assembly of CRL complex. To provide further insights into the nature of this interaction, we performed temperature-dependent ITC titrations and determined a change in heat capacity Δ*C_P_* = −450 cal/mol/K (titration curves shown in Fig. 2*B*). Fig. 2*D* demonstrates a plot with a temperature-dependent change of thermodynamic parameters of SOCS2-EloBC/Cul5_NTD_ interaction. The experimental Δ*C_P_* value is calculated from the slope of the Δ*H* linear regression. As a comparison, previously reported Δ*C_P_* values for ASB9-EloBC/Cul5_NTD_ and Vif-EloBC/Cul5_NTD_ interactions were found to be −350 cal/mol/K (18) and −300 cal/mol/K (30), respectively.

We next calculated the theoretical solvent-accessible surface area values in GetArea (32) and NACCESS (33) software using the crystal structure of SOCS2-EloBC-Cul5_NTD_ complex (PDB code 4JGH) as a model (12) (Table 2). Theoretical Δ*C_P_* values were calculated using the following equation (34),

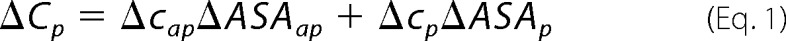
 where Δ*ASA* is the apolar (*ap*) and polar (*p*) surface buried upon interaction of the proteins, and Δ*c* is the area coefficient, representing per Å^2^ contribution of residues in heat capacity change. The polar and non-polar area coefficients represent values empirically determined from a range of protein data sets by different groups (35–39) (reviewed in Ref. 34). We observe good agreement between theoretical and experimental data when using area coefficients according to Refs. 39 and 37 (Table 3).

**TABLE 2:**
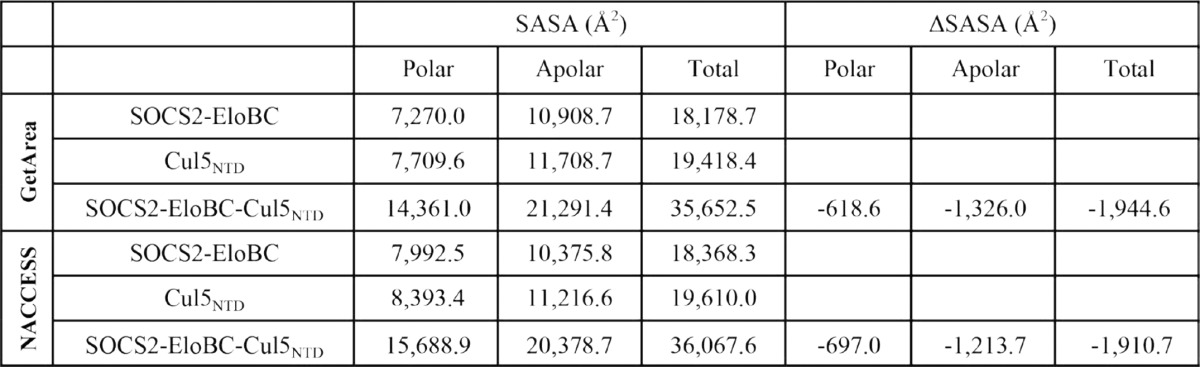
**Large negative Δ*C_P_* value for SOCS2-EloBC/Cul5_NTD_ interaction indicates a highly hydrophobic interface between the proteins; theorectical ΔSASA values for SOCS2-EloBC/Cul5_NTD_ interaction calculated using GetArea and NACCESS programs**

**TABLE 3:**
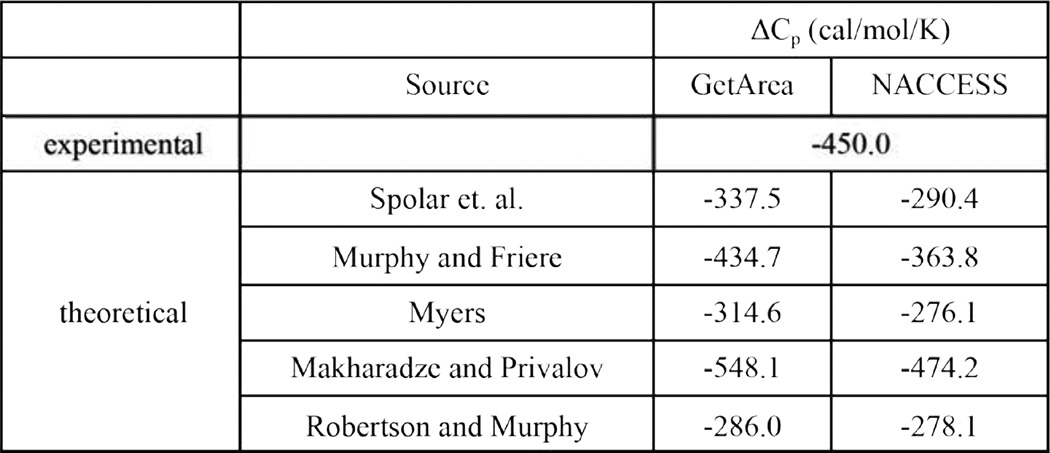
**Large negative Δ*C_P_* value for SOCS2-EloBC/Cul5_NTD_ interaction indicates a highly hydrophobic interface between the proteins; comparison between theorectical and experimental Δ*C_P_* values shows good agreement**

##### SOCS2-EloBC Forms Stable Monomeric Complexes with Cul5_NTD_ and Cul5-Rbx2

**FIGURE 3.**
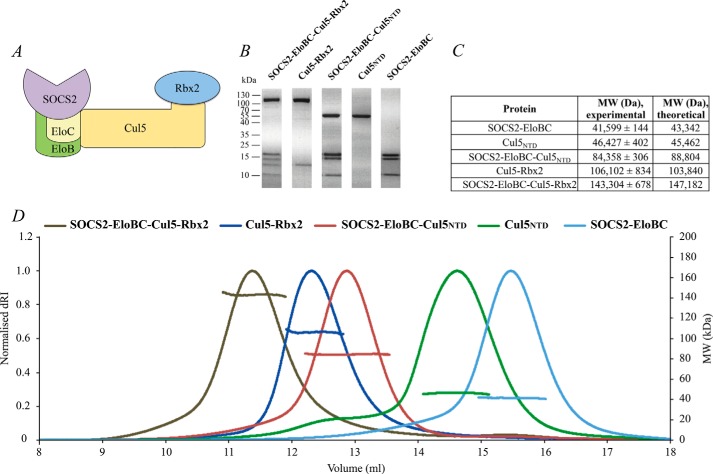
**Recombinant components of CRL5^SOCS2^ assemble into the monomeric full-size protein complex.** *A*, schematic representation of CRL5^SOCS2^, which includes substrate receptor SOCS2, adaptor EloBC complex, scaffold Cul5, and RING domain protein Rbx2. *B*, SDS-polyacrylamide gel images of the purified protein complexes. *C*, *table* showing a comparison of theoretical protein molecular weights against values experimentally determined by SEC-MALS. *D*, SEC-MALS elution profiles for the individual components of CRL5^SOCS2^ and their complexes, including full-size SOCS2-EloBC-Cul5-Rbx2 complex.

To validate formation of CRL5^SOCS2^ and determine the stoichiometry of subunits in the complex, we demonstrated assembly of the full-length E3 ligase *in vitro* using recombinantly expressed and purified protein components (schematic representation in Fig. 3*A*). SOCS2 and EloBC were co-expressed in *E. coli* to obtain the SOCS2-EloBC ternary complex, and Cul5_NTD_ was independently expressed in *E. coli*. The Cul5-Rbx2 protein complex was co-expressed in Sf21 insect cells.

**FIGURE 4.**
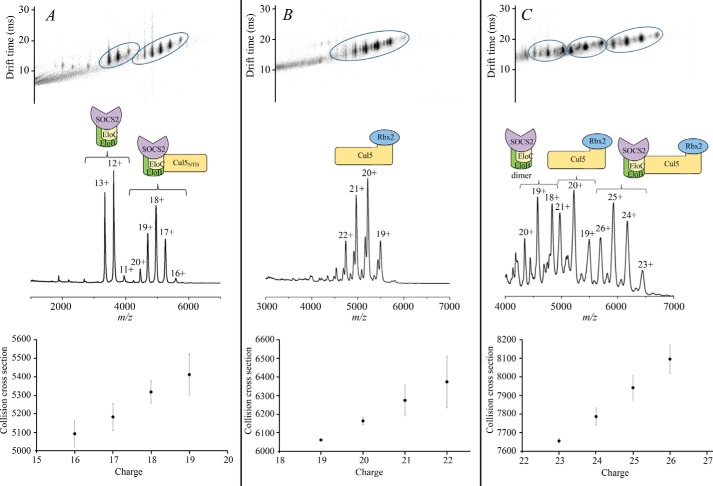
**Ion mobility drift time plot (*top*), corresponding native mass spectra (*middle*), and collision cross-sections (*bottom*) for the CRL5^SOCS2^ complexes and their components.** *A*, SOCS2-EloBC-Cul5_NTD_; *B*, Cul5-Rbx2; *C*, full-size complex SOCS2-EloBC-Cul5-Rbx2. *Error bars*, S.D.

**FIGURE 5.**
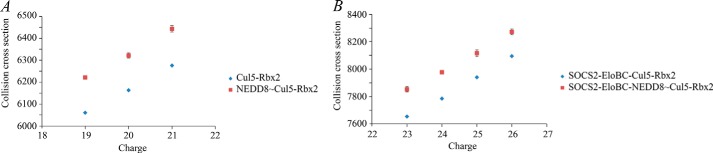
**Experimental ion mobility data for a range of charge states suggests an increase in CCS values upon neddylation of the protein complexes.** Shown are collision cross-sections for Cul5-Rbx2 and NEDD8∼Cul5-Rbx2 (*A*) and SOCS2-EloBC-Cul5-Rbx2 and SOCS2-EloBC-NEDD8∼Cul5-Rbx2 (*B*). *Error bars*, S.D.

**FIGURE 6.**
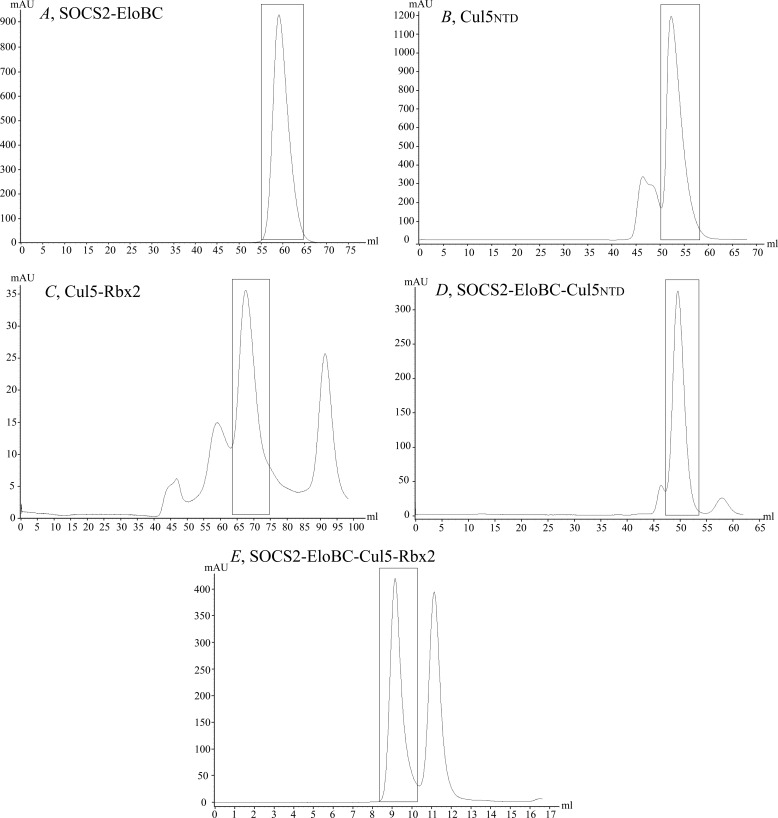
**Gel filtration UV traces.** *A*, SOCS2-EloBC; *B*, Cul5_NTD_; *C*, Cul5-Rbx2; *D*, SOCS2-EloBC-Cul5_NTD_; *E*, SOCS2-EloBC-Cul5-Rbx2. Proteins were purified using multistep purification with size exclusion chromatography as the last step. Peak fractions corresponding to the appropriate proteins were pooled together. The purity and identity of each protein were confirmed using denaturing electrospray ionization-MS (data not shown) and SDS-PAGE.

To characterize the purified protein complexes, biophysical analyses were carried out using SEC-MALS (Fig. 3), native MS, and TWIM-MS techniques (Figs. 4 and 5). SOCS2-EloBC-Cul5_NTD_ and SOCS2-EloBC-Cul5-Rbx2 protein complexes were formed by mixing SOCS2-EloBC and either Cul5_NTD_ or Cul5-Rbx2 components in equimolar amounts and then purified using size exclusion chromatography (Fig. 6).

The SEC-MALS elution profiles of the different protein components and complexes show that they all exist as monomeric and monodisperse entities (Fig. 3*D*). The molar mass over elution peaks is shown in corresponding *colors*. Molecular weight values of eluted proteins are summarized Fig. 3*C*. The results of SEC-MALS analysis confirm the formation of expected protein complexes with experimentally determined molecular weights that correlate well with theoretical values.

##### Experimental Collision Cross-sections for Protein Complexes Are in Good Agreement with Theoretical Values

**FIGURE 7.**
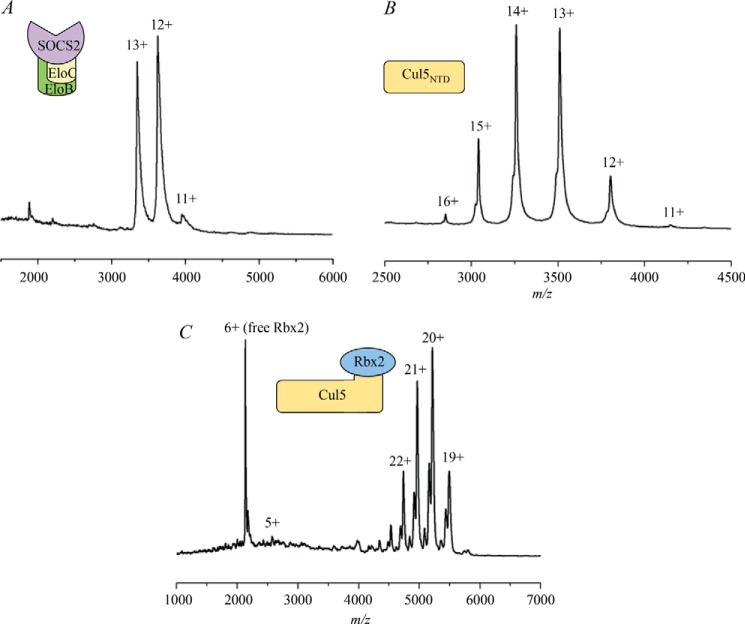
**Native MS spectra.** *A*, SOCS2-EloBC; *B*, Cul5_NTD_; *C*, Cul5-Rbx2.

To validate the structural model of SOCS2-EloBC-Cul5-Rbx2, TWIM-MS was used to examine the molecular weight and stoichiometry of the intact protein complexes as well as to confirm their topology by CCS measurements. The protein components SOCS2-EloBC and Cul5_NTD_ alone were first analyzed using native MS, and the resulting spectra are shown in Fig. 7, *A* and *B*. The masses were confirmed as ∼43 and 45 kDa, respectively. Theoretical and experimental masses for each complex are shown in Table 4.

**TABLE 4:**
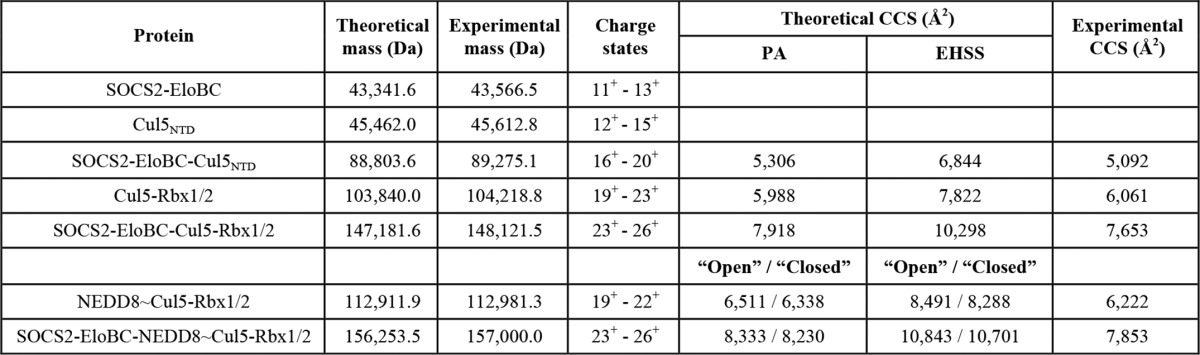
**Summary of the experimental and theorectical CCS data** Shown are masses measured for the protein complexes and observed charge state ranges. Shown is a comparison of experimental CCS values of the protein complexes *versus* their theoretical values calculated using the PA and the EHSS methods. Presented data also include calculated theoretical CCS values for “open” and “closed” NEDD∼Cul5-Rbx1 and SOCS2-EloBC-NEDD∼Cul5-Rbx1 complexes.

Combining SOCS2-EloBC with Cul5_NTD_ produced an 89-kDa complex, which could be detected with charge states ranging from 16+ to 20+ (Fig. 4*A*). Some free SOCS2-EloBC was also observed in this spectrum, with the same charge states as the native mass spectrum of SOCS2-EloBC alone, indicating that some dissociation in solution occurs (Fig. 7*A*). Table 4 shows the experimental CCS values compared with the theoretical ones calculated with the PA and the EHSS methods. It is normally expected that the experimental values would be smaller than the EHSS results and larger than the PA results (24, 25). The collision cross-section determined using ion mobility for SOCS2-EloBC-Cul5_NTD_ is 5,092 Å^2^ for the most native charge state (16+; Fig. 4*A*, *bottom*), which is reasonably close to the theoretical value calculated for the model (Table 4, PA value 5,306 Å^2^).

A typical spectrum of the Cul5-Rbx2 complex is shown in Fig. 7*C* with a predominant 6+ charge state for Rbx2 and a series of charge states from 19+ to 22+ representing the binary complex (104 kDa). Fig. 4*B* shows the Cul5-Rbx2 complex in more detail in addition to the drift time plot. Moreover, there are less intense peaks to the left-hand side of the predominant peak corresponding to a loss of ∼1 kDa from the complex that may represent a truncation in the Cul5 subunit. These species are clearly separated, however, by their ion mobility (Fig. 4*B*), so it is possible to calculate a collisional cross-section for the intact complex. The CCS value from these data for the most native 19+ charge state was found to be 6,061 Å^2^ (Fig. 4*B*, *bottom*), which compared well with the theoretical value (Table 4, PA value 5,988 Å^2^).

The native mass spectrum of the SOCS2-EloBC-Cul5-Rbx2 showed intense peaks at 3,000–4,000 *m*/*z*, indicating a relative abundance of free SOCS2-EloBC, with charge states 11+ to 13+, as described previously (Fig. 7*A*). It is possible that there is an excess of SOCS2-EloBC in these samples or that this subunit has a greater ionization efficiency compared with the other protein components. Fig. 4*C* (*bottom*) depicts the 4,000–7,000 *m*/*z* range of the SOCS2-EloBC-Cul5-Rbx2 spectrum that shows peaks ranging from 4,300 to 4,900 *m*/*z* representing a small amount of SOCS2-EloBC dimer. Second, at 4,700–5,600 *m*/*z*, the Cul5-Rbx2 complex is detected with charge states from 19+ to 22+. Finally, the peaks representing the full 148-kDa complex, SOCS2-EloBC-Cul5-Rbx2, are in the range of 5,600–6,600 *m*/*z*, with charge states 23+ to 26+.

The CCS values measured for each charge state of the full 148-kDa complex are displayed in Fig. 4*C* (*bottom*). For the lowest charge state of SOCS2-EloBC-Cul5-Rbx2, an experimental cross-section of 7,653 Å^2^ was determined compared with a theoretical CCS of 7,918 Å^2^ (Table 4), confirming the structural model as shown in Fig. 10*D*. In this case, the experimentally determined value is slightly smaller than the theoretical value, which could indicate that the structure is slightly more compact than the model suggests.

**FIGURE 8.**
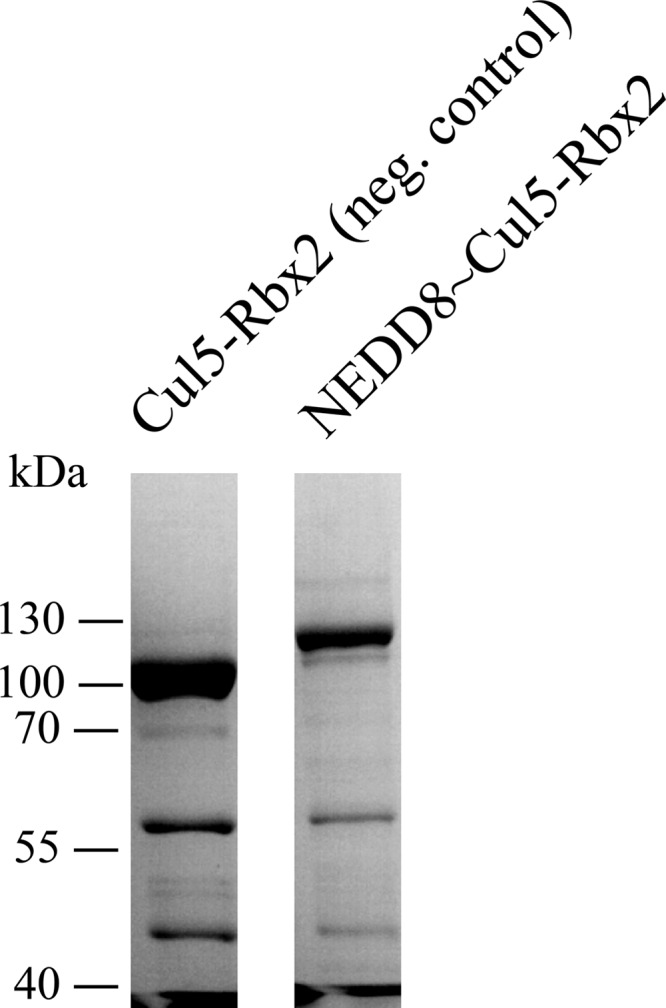
**SDS-polyacrylamide gel images of the neddylation reaction products.** No neddylated product was observed in the negative control reaction (*left*). *Right*, NEDD8∼Cul5-Rbx2.

**FIGURE 9.**
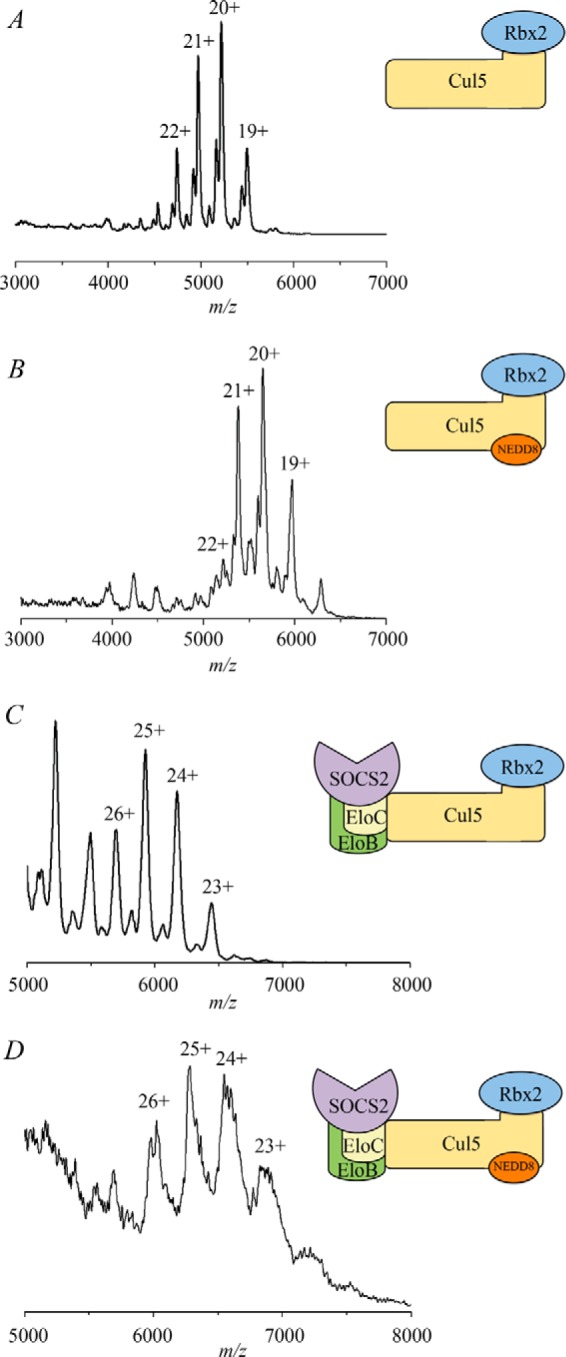
**Comparison of native MS spectra.** *A*, Cul5-Rbx2; *B*, NEDD8∼Cul5-Rbx2; *C*, SOCS2-EloBC-Cul5-Rbx2; *D*, SOCS2-EloBC-NEDD8∼Cul5-Rbx2.

**FIGURE 10.**
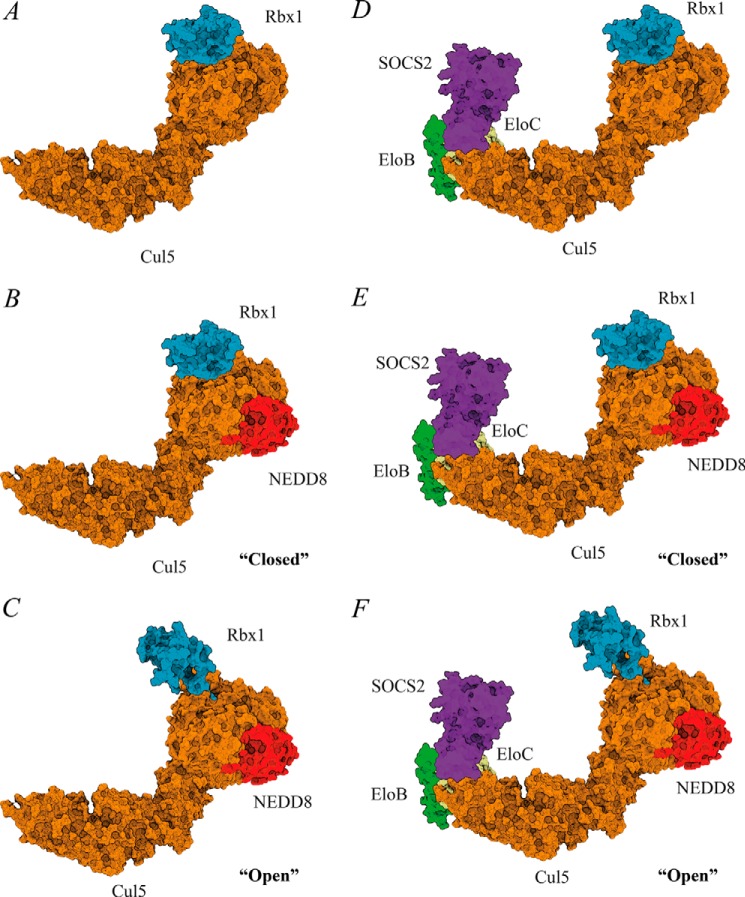
**Structural models provide important insights into the assembly and architecture of CRL5^SOCS2^.** *A*, Cul5-Rbx1; *B*, NEDD8∼Cul5-Rbx1 (closed model); *C*, NEDD8∼Cul5-Rbx1 (open model); *D*, SOCS2-EloBC-Cul5-Rbx1; *E*, SOCS2-EloBC-NEDD8∼Cul5-Rbx1 (closed); *F*, SOCS2-EloBC-NEDD8∼Cul5-Rbx1 (open). The models were assembled using available crystal structures. Due to the lack of Rbx2 crystal structure, its closest homolog, Rbx1, was used instead. These models were used for calculation of the theoretical CCS values.

To investigate the effect of neddylation on the complex assembly, we performed *in vitro* neddylation assays on the purified Cul5-Rbx2 complex (Fig. 8) and used the reaction product NEDD8∼Cul5-Rbx2 to reconstitute neddylated full complex SOCS2-EloBC-Cul5-Rbx2. The masses for neddylated complexes (Table 4, 113 and 157 kDa, respectively) are in agreement with the theoretical mass for the addition of NEDD8. First, the same range of charge states was observed for the non-neddylated and neddylated complexes in the native mass spectra (Fig. 9). This would indicate that no significant conformational rearrangement had occurred. Second, the CCS values measured by TWIM-MS for each charge state of the neddylated complexes were compared with those of the non-neddylated ones (Fig. 5, *A* and *B*), showing an increase in CCS of 150–200 Å^2^ in each case (Table 4). We compared the experimental data with two alternative models: one “open” model that assumes a conformational change upon neddylation, as observed crystallographically for NEDD8∼Cul5_CTD_-Rbx1 (27), and a second “closed” model that simply has NEDD8 added onto the non-neddylated complex without any conformational rearrangement, with the aim to distinguish them based on the TWIM-MS data. The calculated CCS increase for the two alternative neddylated models (NEDD8∼Cul5-Rbx1) *versus* the non-neddylated one (Fig. 10, *A–C*), is 350 Å^2^ for the closed model and 523 Å^2^ for the open model (PA method; Table 4), whereas for SOCS2-EloBC-NEDD8∼Cul5-Rbx1, the neddylation accounts for an extra 312 Å^2^ (closed) and 415 Å^2^ (open). It would therefore appear that the increase in size is predominantly due to the addition of NEDD8 rather than a significant conformational change.

## DISCUSSION

Here, we show that all CRL5^SOCS2^ components SOCS2, EloBC, Cul5, and Rbx2 can be specifically pulled down from the human cell lysates with subsequent validation of their identity by MS analysis. These components were recombinantly expressed and purified and then assembled *in vitro* into different sized complexes up to the full-size E3 ligase.

Biophysical studies of the full-size CRLs are important for better understanding the principles of assembly and to gain insight into their structural architecture. This is particularly relevant for the cases where crystal structures are not available, as for CRL5^SOCS2^. We addressed this by presenting the first report of *in vitro* assembly of full-size human CRL5^SOCS2^ reconstituted from recombinant components and provide a biophysical analysis of the obtained complexes.

The structural model of the SOCS2-EloBC-Cul5-Rbx2 complex was validated by TWIM-MS studies. The experimentally measured CCS values are in agreement with the theoretically calculated ones, although the molecular architecture of SOCS2-EloBC-Cul5_NTD_ and SOCS2-EloBC-Cul5-Rbx2 appears to be slightly more compact than predicted.

Modification of the cullin scaffold with NEDD8 protein is crucial for the activation of CRLs (40). Previous structural studies showed that NEDD8 promotes a conformational rearrangement of the Cul5-Rbx1 component of CRL5 (27). Such a structural alteration enables Rbx1-E2∼ubiquitin to extend toward the substrate receptor subunit, thereby promoting substrate polyubiquitination. One of the proteins identified in the pull-down experiments was NEDD8, supporting the presence of the active neddylated complex inside cells. Comparison of TWIM-MS data between neddylated and non-neddylated Cul5-Rbx2 and SOCS2-EloBC-Cul5-Rbx2 complexes showed an increase of 150–200 Å^2^ in CCS values (Table 4). Interestingly, the increase in calculated CCS values defined by the addition of NEDD8 is ∼2–3 times larger than the conformational change of the Rbx1 subunit in the open models (Fig. 10, *C* and *F*). Therefore, the difference between open and closed neddylated models is not significant enough, and we cannot distinguish them based on the experimental data. The observed change in CCS is therefore largely due to the addition of the extra NEDD8 subunit.

One of the main limitations of biophysical studies of the whole multisubunit CRLs is the difficulty of obtaining all of the components in appropriate amount and quality, particularly full-length cullins. Expression and purification of stable full-length cullin scaffolds in complex with RING domain proteins is not trivial and has been previously reported only for Cul1-Rbx1 (26), Cul4A-Rbx1 (41), Cul4B-Rbx1 (42), and Cul5-Rbx2 (20). As a result, there were only a few cases in the literature describing characterization of the full-size CRLs assembled from recombinant subunits (26, 42). To purify the Cul5-Rbx2 complex in this study, we used the Dac tag technology, which provides additional stability and solubility to the protein complex and additionally improves the yield of recombinant proteins (19). This approach has also proven to be successful for purification of Cul2-Rbx1 complex^4^ and could be further extended to other cullins and large multisubunit complexes.

In certain cases, CRLs exist and function in homo- or hetero-oligomeric states. The biological implications of CRL oligomerization are postulated to include activity regulation, enhancement of substrate ubiquitination, and alternative mechanistic aspects of ubiquitin transfer (6). For example, several studies have shown that CRL3 can dimerize via an adaptor BTB domain (43) or through NEDD8-mediated interaction between two Cul3 scaffolds (44). CRL1 was also demonstrated to be able to dimerize via the receptor Cdc4 (cell division control protein 4), resulting in enhanced ubiquitination of substrate Sic1 (45). Additional examples include other BTB receptor/adaptor subunits of CRL3 (46–49), F box receptors of CRL1 (45, 50–53), and the DCAF receptor of CRL4 (54). More recently, a two-site model for substrate recognition was proposed for CRL3^KLHL11^ based on the crystal structure of KLHL11-Cul3_NTD_ (49). However, no evidence for dimerization of elongins, cullin 2 or cullin 5, or SOCS subunits has been reported to date. In this work, using SEC-MALS and native MS techniques, we have established that CRL5^SOCS2^ exists in a monomeric state. We provide a structural model validated by TWIM-MS studies that suggests a similar mechanism of ubiquitin transfer to a previously reported monomeric CRL1^Skp2^ complex (26). Therefore, according to the proposed model, CRL5^SOCS2^ oligomerization does not seem to be necessary for enzyme activity.

Our measured *K_d_* values for the interaction of SOCS2-EloBC with GHR_pY595 peptide or Cul5_NTD_ are in good agreement with previously reported data (11, 31). Weak SOCS2-EloBC/GHR_pY595 interaction (*K_d_* = 1.8 μm) could suggest low selectivity toward a particular substrate and instead the ability to target a variety of phosphorylated proteins. In contrast, the interaction of SOCS2-EloBC with scaffold Cul5_NTD_ is very tight (*K_d_* = 11 nm at 298 K). The large negative Δ*C_P_* value (−450 cal/mol/K) for the SOCS2-EloBC/Cul5_NTD_ interaction indicates a major contribution of the hydrophobic interface and further reflects the high affinity (34, 38) (*e.g.* when compared with other related interactions, such as ASB9-EloBC/Cul5_NTD_ and Vif-EloBC/Cul5_NTD_) (18, 30). Overall, these results indicate the structural importance of the SOCS2-EloBC/Cul5_NTD_ interface for assembly and stability of the CRL5^SOCS2^.

As the next logical step following the current study, we believe it would be important to develop an assay to measure activity of the recombinant CRL5^SOCS2^ against the substrate GHR protein resulting in ubiquitination and the subsequent proteasomal degradation of the latter. Such an assay could be useful for testing the potency of small molecule modulators of CRL5^SOCS2^ activity. In accordance with this, a recent example demonstrates *in vitro* reconstitution of murine CRL5^SOCS3^, containing SOCS3, a close homolog of SOCS2, as a substrate receptor subunit (55). The authors used co-expressed Cul5_NTD_, Cul5_CTD_, and Rbx2 proteins to form a complex with SOCS3-EloBC, and the assembled E3 ligase then demonstrated activity in the ubiquitination assay against substrates JAK2 and gp130.

In addition, it would be important to obtain the crystal structure of the receptor SOCS2 bound to the substrate GHR depicting the details of the interface between these two proteins. This could substantially advance the development of inhibitors of this interaction (*i.e.* structural phosphotyrosine analogs or isosteres). The biophysical insights into the interactions and assembly of the full-size CRL5^SOCS2^ E3 ligase reported in our study will aid future developments in this direction.

## Footnotes

4 A. Knebel, unpublished results.

## Footnotes

3 The abbreviations used are:

CRL: cullin-RING E3 ubiquitin ligase
BTB: bric-a-brac·tram-track·broad complex
CCS: collision cross-section(s)
CRL5^SOCS2^: SOCS2-EloBC-Cul5-Rbx2 complex
CTD: C-terminal domain
Cul5: cullin 5
DCAF: DDB1 and Cul4-associated factor
DDB1: DNA damage-binding protein 1
EloB: elongin B
EloC: elongin C
EloBC: elongin B-elongin C complex
EHSS: exact hard sphere scattering
FBXO31: F box-only protein 31
GHR: growth hormone receptor
TWIM-MS: travelling wave ion mobility-mass spectrometry
ITC: isothermal titration calorimetry
NEDD8: neural precursor cell expressed developmentally down-regulated protein 8
NTD: N-terminal domain
PA: projection approximation
PDB: Protein Data Bank
Rbx: RING box protein
Skp: S-phase kinase-associated protein
SEC-MALS: size exclusion chromatography and multiangle light scattering
SOCS2: suppressor of cytokine signaling 2
TCEP: tris(2-carboxyethyl) phosphine hydrochloride
TEV: tobacco etch virus
SH2: Src homology 2.
